# The effect of cognitive aging on implicit sequence learning and dual tasking

**DOI:** 10.3389/fpsyg.2014.00154

**Published:** 2014-02-27

**Authors:** Jochen Vandenbossche, Daphné Coomans, Koen Homblé, Natacha Deroost

**Affiliations:** ^1^Research Unit for Clinical Experimental Psychology – Klinisch-Experimentele Psychologie, Vrije Universiteit BrusselBrussels, Belgium; ^2^Center for Neurosciences, Vrije Universiteit BrusselBrussels, Belgium

**Keywords:** sequence learning, dual tasking, lifespan, cognitive aging, working memory load, interference

## Abstract

We investigated the influence of attentional demands on sequence-specific learning by means of the serial reaction time task (Nissen and Bullemer, 1987) in young (age 18–25) and aged (age 55–75) adults. Participants had to respond as fast as possible to a stimulus presented in one of four horizontal locations by pressing a key corresponding to the spatial position of the stimulus. During the training phase sequential blocks were accompanied by (1) no secondary task (single), (2) a secondary tone counting task (dual tone), or (3) a secondary shape counting task (dual shape). Both secondary tasks were administered to investigate whether low and high interference tasks interact with implicit learning and age. The testing phase, under baseline single condition, was implemented to assess differences in sequence-specific learning between young and aged adults. Results indicate that (1) aged subjects show less sequence learning compared to young adults, (2) young participants show similar implicit learning effects under both single and dual task conditions when we account for explicit awareness, and (3) aged adults demonstrate reduced learning when the primary task is accompanied with a secondary task, even when explicit awareness is included as a covariate in the analysis. These findings point to implicit learning deficits under dual task conditions that can be related to cognitive aging, demonstrating the need for sufficient cognitive resources while performing a sequence learning task.

## INTRODUCTION

Abstracting and learning regularities in our environment, implicitly or explicitly, is essential for daily functioning. Traditionally, implicit learning is defined as not explicit or as an automatic process in which we learn without intention (Cleeremans et al., 1998). Although the automaticity of implicit learning is supported in studies depleting selective and executive attention in young adults (e.g., Coomans et al., 2011; Deroost et al., 2012), research concerning aged adults is demonstrating heterogeneous results (e.g., Frensch and Miner, 1994; Nejati et al., 2008; Gamble et al., 2014). However, since adults are experiencing more difficulties in cognitive controlled processes when aging, hence cognitive aging, it is important to know whether implicit learning can be categorized as automatic or controlled. Depending on this, implicit learning tasks can be implemented in training programs promoting active aging. For this purpose, we want to study implicit learning by using a dual task paradigm, and additionally investigate whether different secondary tasks, inducing high or low interference with the primary task, yield different results in young and aged adults. This knowledge could enable us to pinpoint implicit learning deficits and consequently conceive practical guidelines with a focus on the empowerment of the elderly, compensating for cognitive decline.

Across lifespan, several cognitive functions are prone to decline, which is often described as *cognitive aging*. Cognitive aging can be characterized as a decrease in or a reduced availability of cognitive resources, acting as a key contributor to age-related decline in a range of cognitive tasks (Park and Schwarz, 2000). Deficits are generally situated in executive functioning, memory and learning tasks, as well as higher order processes of language and intellectual competence. For example, explicit learning is frequently reported to be impaired in the elderly (Hedden and Gabrieli, 2004). In contrast, implicit learning is expected to be kept relatively preserved with age (Howard and Howard, 1992; Midford and Kirsner, 2005; Gaillard et al., 2009; Kürten et al., 2012), although diminished and impaired learning has been observed when task requirements are made more difficult (Curran, 1997; Howard and Howard, 1997).

When investigating implicit learning under dual task conditions, a secondary task is only added to the training phase of the implicit learning task, e.g., the serial reaction time task (SRT task; Nissen and Bullemer, 1987), to manipulate working memory load without interfering with the expression of learning (Frensch et al., 1998). In the SRT task, subjects need to respond as fast as possible to a stimulus presented in one of four locations by pressing a key corresponding to the spatial position of the stimulus. Unbeknown to the participants, the order in which the stimulus appears follows a repeating sequence. Typical results show that reaction times (RTs) decrease progressively over training (general training effect), and increase significantly when the location sequence is replaced by a random order sequence (sequence learning). The key question in the present study is whether the depletion of cognitive resources hampers implicit learning in aged adults. Assuming this being the case, we expect that implicit learning will be impaired under dual task conditions as the secondary task depletes the pool of cognitive resources, thereby leaving less capacity to learn sequential knowledge in the primary task. In aged subjects, we expect a larger impact of the dual task paradigm for aging is associated with limited cognitive resources (Park and Schwarz, 2000).

A review of the literature shows that the SRT task in combination with a secondary task has not been massively investigated in aged adults. Frensch and Miner (1994, see Experiment 3) demonstrated that aged adults show specific impairments under dual task conditions (dual tone task), hence illustrating the influence of cognitive aging on implicit learning. More recent, Nejati et al. (2008) confirmed these results and postulated that learning cannot take place under attention-based conditions (dual tone task) in aged adults. In contrast, Gamble et al. (2014) asked participants to memorize items in advance (matrices) and keep them in memory while completing a sequenced block of trials. They demonstrated that implicit learning is rather independent of the availability of cognitive resources as memory load only affected aged adults by suppressing the expression of learning, not learning itself.

In the current research, we aim to determine whether the level of *interference *has an impact on**learning under dual task conditions. For example, counting tones while performing the primary task can introduce inconsistencies in the succession of sequenced trials, leading to a temporal disruption of the sequence (Stadler, 1995). In addition, the inclusion of random stimuli during the response-stimulus interval (RSI) acts as noise and can impair implicit learning under dual task conditions. To this extent, we added a low (SRT task combined with a standard tone counting task) and a high (presentation of secondary task stimuli within the primary task, e.g., triangle instead of target dot) interference condition to the SRT task. Although the tone counting task is perceived as highly disruptive (Jiménez and Méndez, 1999; Jiménez and Vazquez, 2005), the shape counting task causes possibly even more interference as participants were required to (1) count distracters presented in the same modality as the target (visual), as opposed to the tone counting task where different modalities were used (visual-auditory causing less interference; Duncan et al., 1997), and (2) count items in parallel with the presentation of the target (shape) instead of after responding to the location of the target (tone).

In sum, we expect that implicit learning will be affected under dual task conditions in the elderly. Moreover, we conjecture that implicit learning will be more impaired under high interference (dual shape) compared to low interference (dual tone) as less cognitive resources will be available to learn in the primary task. These results could extend the findings of Frensch et al. (1998), claiming that implicit learning is impaired according to different interference levels in aged adults. In young adults, however, we expect that learning under single and dual task conditions will be highly comparable as their cognitive resources are not affected by cognitive aging processes.

## MATERIALS AND METHODS

### PARTICIPANTS

#### Young

Forty-five students of the Vrije Universiteit Brussel (VUB), each appointed randomly to one of three test conditions, executed the experiment in return for course credit of an introductory psychology course. Fifteen students (four men, mean age = 19.4 years, SE = 0.24, range = 18–21) were included in the SRT single task condition, 15 young adults (four men, mean age = 18.6 years, SE = 0.47, range = 18–25) performed the SRT dual tone, and 15 subjects (six men, mean age = 21.4 years, SE = 0.65, range = 18–28) were assigned to the SRT dual shape condition.

#### Aged

Forty-five healthy aged adults were randomly divided into three equal samples matched for age (*n* = 15) and each sample was assigned to a specific test condition (single, dual tone, dual shape). In the single task condition, age ranged from 57 to 76 years (nine men, mean age = 66.1 years, SE = 1.45). In the dual tone condition, age ranged from 57 to 81 years (11 men, mean age = 66.3 years, SE = 1.93), and in the dual shape condition, age ranged from 55 to 78 years (11 men, mean age = 63.7 years, SE = 1.65). By means of self-report, we only included participants that (1) were in general good health without history of depression, motor dysfunction or neurological disorders, and (2) had normal to corrected-to-normal vision. Participation in the experiment was voluntarily with informed consent in accordance with the Ethics Committee of the VUB.

### DESIGN AND PROCEDURE

Experiments were conducted individually in semi-darkened cubicles of the psychological laboratory of the VUB. The SRT task was run on a Pentium four computer with a 17 inch screen, using E-prime Version 1.1 software (Schneider et al., 2002). In all three SRT conditions (single, dual tone, and dual shape), implicit learning of a deterministic first-order conditional (FOC) sequence was tested to minimize the potential age differences in the single task condition: 132342134142 (used in single condition) and 241431243231 (used in both dual tone and dual shape conditions), the numbers 1–4 denote the leftmost, left, right, and rightmost target position, respectively. Both sequences were structurally identical (note that the second sequence was created by replacing position 1 with 2 and 3 with 4 in the first sequence) so that possible differences in sequence learning could not be attributed to differences in sequence structure. The FOC sequence was continuously repeated over the experimental Blocks 1–12. During Block 11, the sequence was presented in a random order to assess sequence learning. The random sequence introduced in Block 11 was generated on the basis of a random seed that differed between participants. The four stimulus alternatives occurred equally often in all structured and random sequences.

#### SRT single

Participants were instructed to react to the location of the target dot, as fast and as accurately as possible. Four horizontally aligned white squares of side 1.5 cm (or 1.4° visual angle) were presented against a light gray background. These squares remained on screen throughout a block of trials. Gaps between two squares measured 2.5 cm (or 2.4° visual angle with a viewing distance of approximately 60 cm). On each trial, a black dot of 8 mm diameter (or 0.8° visual angle) appeared in one of four squares. The “c,” “v,” “b,” and ”n” keys, situated on the bottom row of an AZERTY keyboard, corresponded to a leftmost, left, right, and rightmost target and had to be pressed with the left middle finger, left index finger, right index finger, and right middle finger, respectively.

First, one practice block of 50 trials in random order was given to train the stimulus-response mapping. After practice, subjects performed 12 experimental blocks of 72 trials. In the beginning of each block, a warning signal for the upcoming trials appeared, requiring participants to rest their fingers lightly on the four response keys. The target was presented until the response was made. Subsequently, after an RSI of 50 ms, the next target appeared. This was shorter compared to the study of Frensch et al. (1998; RSI 200 ms) to diminish explicit awareness (Destrebecqz and Cleeremans, 2001). RTs and accuracy measures were recorded for each trial. In case of an incorrect response, the word “Error” was presented in Dutch for 750 ms. No error corrections were possible. After each block of trials, patients received feedback about their RTs and error rates for that particular block. A break of 30 s was imposed before the next block started.

#### SRT dual tone

The procedure for the SRT dual tone task is largely similar to the one used in the SRT single task. During the training phase (practice block and experimental Blocks 1–9), however, a secondary counting task accompanied the SRT procedure. Participants were asked to keep track of the number of “gunshots” they heard among irrelevant low-pitched tones (1000 Hz). After each block, they were required to report the perceived number of shots they counted during this block. Both shots and tones had a duration of 50 ms, and were provided through a headphone. The shots or tones were presented in the time interval between the participant’s response to the target dot and the presentation of the next target (50 ms RSI), and varied between 24 and 30 times per block. We used gunshots instead of common high-pitched tones in order to increase stimulus contrast. Participants were encouraged to count gunshots as accurately as possible. Since the presence of a secondary task might only be suppressing the expression of implicit learning, the counting task was no longer presented during the testing phase (Blocks 10, 11, and 12; Frensch et al., 1998).

#### SRT dual shape

The procedure for the SRT dual shape task is comparable to the SRT dual tone task, with the only difference that instead of gunshots, appearing triangles had to be counted for each block during the training phase. Contrary to the tones which were presented between target stimuli, triangles occurred within the target stimulus by occasionally replacing the target dot in the task. The number of presentations were similar compared to the tone counting task. Like in the SRT dual tone task, the secondary counting task was not longer presented during the testing phase (Blocks 10, 11, and 12).

#### Awareness questionnaire

At the end of the experiment, we administered a standardized questionnaire to assess awareness of the sequence. A measure to quantify awareness (percentage of the entire sequence correctly reproduced) was obtained by posing general experiment-related questions (“What is the goal of the experiment?,” “Did you notice something particular?”), which gradually turned into more specific sequence-related questions (“Did the target dots appear randomly on the screen, or was there a regularity involved?,” “Was this regularity present in all of the blocks?,” “When you noticed a regularity, try to describe it as precise and accurate as possible?”).

## RESULTS

Univariate and mixed factorial analysis of variances (ANOVAs; with Huyhn–Feldt corrections for violations of sphericity) were implemented to analyze main and interaction effects in general error rates, training, and sequence learning between task conditions and groups. In case of significant differences, Bonferroni *post hoc* tests or planned contrasts were performed. Data analysis was performed using SPSS Version 17.0 and all analyses were two-tailed, using a significance level of 0.05. Differences showing a significance level of 0.10 will be reported as a tendency.

RT analysis was performed on participants’ median RTs of correct trials, with the exclusion of practice trials. Erroneous responses and responses following an error were discarded from the analysis. Error rate analyses did not contradict RT findings, although, in some cases, error rates showed no significant effect where RTs did. However, since error rates are less informative estimates of learning performance compared to RTs, results will focus on the RT data only. In addition, z-scores were calculated for all conditions (Faust et al., 1999), as we observed that raw RTs were significantly faster for young [Single (*M *= 316 ms, SD**= 40 ms), dual tone (*M *= 383 ms, SD**= 64 ms), and dual shape (*M *= 426 ms, SD**= 63 ms)] compared to aged adults [Single (*M *= 490 ms, SD**= 79 ms), dual tone (*M* = 595 ms, SD = 149 ms), and dual shape (*M* = 573 ms, SD = 93ms)], *F*(1,84) = 87.51, *p *= 0.01, $\eta_{p}^{2}$ = 0.98^1^.

### GENERAL ERROR RATES

The absence of significant negative correlations between RTs and error rates showed no indication for speed-accuracy trade-offs under single condition (*r *= -0.45, *p *= 0.10 and *r *= -0.16, *p *= 0.58), dual tone condition (*r *= 0.03, *p *= 0.93 and *r *= -0.10, *p *= 0.71), nor under dual shape condition (*r *= 0.49, *p *= 0.06 and *r *= 0.43, *p *= 0.11) for young and aged subjects, respectively.

A univariate ANOVA with Task Condition and Group as between-subjects factors and mean overall error rate as dependant factor, revealed no significant main effects [main effect Task Condition: *F*(2,84) = 1.14, *p* = 0.47, $\eta_{p}^{2}$ = 0.53; main effect Group *F*(1,84) = 2.94, *p* = 0.23, $\eta_{p}^{2}$ = 0.60]. The Task Condition × Group interaction, however, showed a tendency toward significance, *F*(2,84) = 2.35, *p* = 0.10, $\eta_{p}^{2}$ = 0.05, indicating that the mean overall error rate between task conditions differed more in younger adults [Young: single (4.42%, SE**= 0.61), dual tone (2.94%, SE**= 0.36), and dual shape (2.59%, SE**= 0.46); Aged: single (2.18%, SE**= 0.54), dual tone (2.82%, SE**= 0.50), and dual shape (1.74%, SE**= 0.46)]. A Bonferroni *post hoc* test showed that young adults made more errors under single task condition compared to dual shape condition,* p* < 0.05. Possibly, the instruction to be accurate on a secondary task urges young subjects to focus more on accuracy in general, whereas under single task condition a good performance is subjectively more associated with fast responses instead of more accurate ones.

### SEQUENCE AWARENESS QUESTIONNAIRE

Administering a sequence awareness questionnaire at the end of the SRT task could give us more information on whether participants were explicitly aware of the sequence. Unfortunately, awareness questionnaire data of 10 out of 45 aged (five in the single condition and five in the dual tone condition) and 5 out of 45 young subjects (five in the single condition) were lost. However, still a majority of subjects filled in the questionnaire and therefore, it is still useful to determine how good participants could reproduce the entire sequence correctly. We calculated scores on the post-test questionnaire, reflected in percentages (when predicting all 12 positions correctly, a score of 100% was given), and a high percentage indicates a high chance that subjects were aware of the sequence. A univariate ANOVA with Task Condition (single, dual tone, and dual shape) and Group (young and aged adults) as between-subjects factors, and explicit awareness as dependent factor, revealed a significant interaction effect of Task Condition × Group, *F*(2,73) = 6.78, *p* < 0.01, $\eta_{p}^{2}$ = 0.16. Bonferroni *post hoc* tests were used to compare conditions and indicated significant more explicit awareness (1) in young compared to aged adults under single task conditions (*p* < 0.01), and (2) in single compared to dual tone (*p* < 0.001) and dual shape conditions (*p* < 0.001) in young adults [Aged: single (*M* = 4.17%, SE = 2.85), dual tone (*M* = 4.63%, SE = 3.14), and dual shape (*M* = 5.56%, SE = 3.22); Young: single (*M* = 24.45%, SE = 6.58), dual tone (*M* = 0.00%, SE = 0.00), and dual shape (*M* = 1.67%, SE = 1.67)]. In addition, there was a significant correlation between the questionnaire scores and z-transformed RT sequence learning (random Block 11 versus the mean of Blocks 10 and 12), indicating that learning performance was positively determined by sequence awareness (*r* = 0.36, *p* = 0.001).

Due to the perceived high level of explicit intrusion under single task conditions in young adults, it could be a concern that explicit knowledge acts as a confounder in sequence learning. Explicit intrusion, however, is less likely to occur under dual tasking, thereby explaining general diminished learning compared to a single task condition (Cleeremans et al., 1998). To account for explicit intrusion, we will therefore investigate training and sequence learning effects by carrying out analysis of covariances (ANCOVAs) with explicit awareness (score on the post-test questionnaire) as a covariate.

### TRAINING EFFECTS

General training effects are indicated by a decrease in z-transformed RTs across experimental Blocks 1–9. To estimate training effects, we carried out a repeated measures ANCOVA with Task Condition (single, dual tone, and dual shape) and Group (young and aged adults) as between-subjects factors, Training (Blocks 1–9) as within-subjects factor, and Explicit Awareness as a covariate.

As expected, the main effect of Group was not significant since RTs were z-transformed in order to account for baseline RT differences, *F*(1,72) = 0.08, *p* = 0.78, $\eta_{p}^{2}$ < 0.01. Consequently, nor the main effect of Task Condition, nor the Task Condition × Group interaction effect reached significance, respectively *F*(2,72) = 0.37, *p* = 0.70, $\eta_{p}^{2}$ = 0.01 and *F*(2,72) = 1.14, *p* = 0.33, $\eta_{p}^{2}$ = 0.03.

**FIGURE 1:**
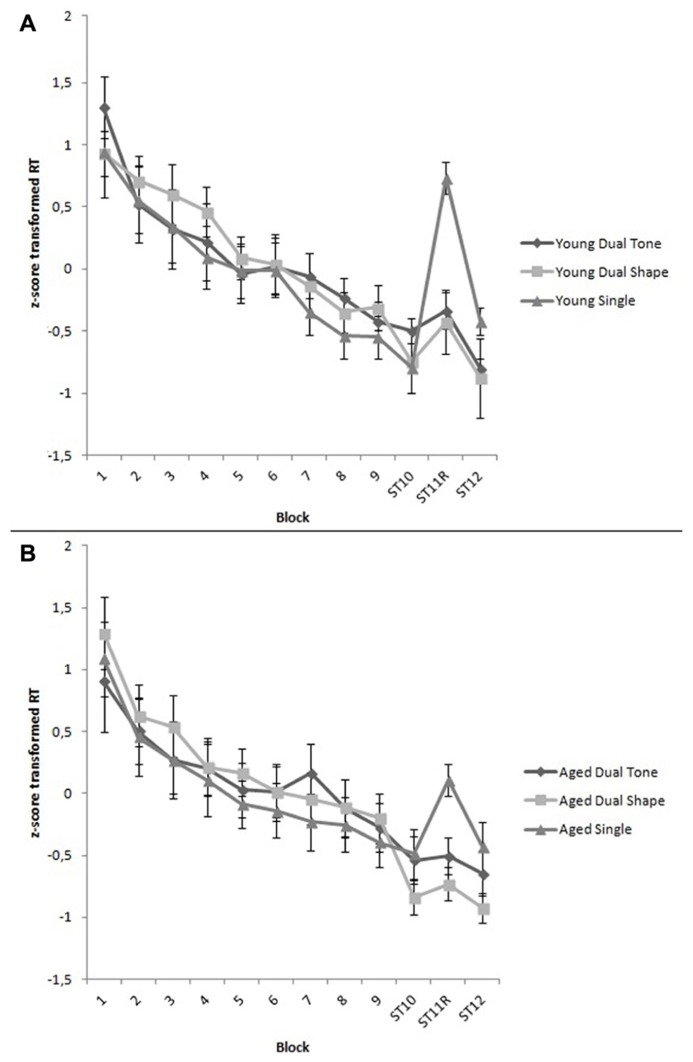
**(A)** z-score transformed RTs per block for young adults under single, dual tone and dual shape conditions. All blocks are structured, except for random Block 11. During the first nine blocks, a secondary counting task was presented in the dual tone and dual shape conditions. Vertical bars denote standard errors. **(B)** z-score transformed RTs per block for aged adults under single, dual tone and dual shape conditions. All blocks are structured, except for random Block 11. During the first nine blocks, a secondary counting task was presented in the dual tone and dual shape conditions. Vertical bars denote standard errors.

The covariate, Explicit Awareness, tended to be related to Training, *F*(4.01,288.82) = 2.61, *p* = 0.04, $\eta_{p}^{2}$ = 0.05. The main effect of Training was significant, *F*(4.01,288.82) = 58.85, *p* < 0.001, $\eta_{p}^{2}$ = 0.45, indicating that RTs generally declined through the experimental blocks. However, the Group × Training, the Task Condition × Training, and the Group × Task Condition × Training interaction effects were not significant, demonstrating that RTs declined evenly across age groups and task conditions, respectively *F*(4.01,288.82) = 1.05, *p* = 0.38, $\eta_{p}^{2}$ = 0.01, *F*(8.02,288.82) = 0.61, *p* = 0.77, $\eta_{p}^{2}$ = 0.02, and *F*(8.02,288.82) = 0.95, *p* = 0.48, $\eta_{p}^{2}$ = 0.03 (see **Figure 1**).

### TRANSFER FROM TRAINING TO TEST PHASE

To give us more insight into the dual task performance in young and aged adults, we ran a repeated measures ANOVA with Task Condition (dual tone and dual shape) and Group (young and aged adults) as between-subjects factors, and Transfer (dual task Block 9 vs. single task Block 10) as within-subjects factor on z-transformed RTs.

Due to z-score transformation, the main effect of Group was not significant, *F*(1,56) = 0.03, *p* = 0.86, $\eta_{p}^{2}$ < 0.01. The main effect of Task Condition and the Group × Task Condition interaction were also not significant, respectively *F*(1,56) = 0.31, *p* = 0.58, $\eta_{p}^{2}$ < 0.01 and *F*(1,56) = 0.02, *p* = 0.90, $\eta_{p}^{2}$ < 0.01*. *Crucially, the main effect of Transfer, and the interaction effects of Transfer × Group and Transfer × Task Condition were significant, respectively *F*(1,56) = 52.20, *p* < 0.001, $\eta_{p}^{2}$ = 0.48, *F*(1,56) = 4.15, *p* = 0.05, $\eta_{p}^{2}$ = 0.07 and *F*(1,56) = 13.97, *p* < 0.001, $\eta_{p}^{2}$ = 0.20. This shows that the transfer effect from dual to single task conditions was larger for (1) aged compared to young adults, and (2) dual shape compared to dual conditions. The Transfer × Group × Task Condition interaction effect was not significant (*F* < 1).

### SEQUENCE LEARNING

Sequence learning is derived from a slowed performance in random Block 11 compared to the mean of the adjacent sequenced Blocks 10 and 12. A repeated measures ANCOVA was conducted with Task Condition (single, dual tone, and dual shape) and Group (young and aged adults) as between-subjects factors, Sequence Learning (random Block 11 versus the mean of Blocks 10 and 12) as within-subjects factor, and Explicit Awareness as a covariate.

Due to z-score transformation the main effect of Group was not significant, *F*(1,72) < 0.01, *p* = 0.98, $\eta_{p}^{2}$ < 0.01. In addition, the main effect of Task Condition and Group × Task Condition interaction were not significant, respectively *F*(2,72) = 0.23, *p* = 0.80, $\eta_{p}^{2}$ = 0.01 and *F*(2,72) = 0.57, *p* = 0.57, $\eta_{p}^{2}$ = 0.02*.*

The covariate, Explicit Awareness, was significantly related to Sequence Learning, *F*(1,72) = 6.54, *p* = 0.01, $\eta_{p}^{2}$ = 0.08. More important was that the main effect of Sequence Learning and the Sequence Learning × Group interaction effect were significant, which indicated that young adults acquired more sequence learning than aged adults, respectively *F*(1,72) = 31.32, *p* < 0.001, $\eta_{p}^{2}$ = 0.30 and *F*(1,72) = 6.70, *p* = 0.01, $\eta_{p}^{2}$ = 0.09. The Sequence Learning × Task Condition interaction effect showed a tendency toward significance, *F*(2,72) = 2.54, *p* = 0.09, $\eta_{p}^{2}$ = 0.07. The Group × Task Condition × Sequence Learning interaction effect was also not significant, *F*(2,72) = 1.05, *p* = 0.36, $\eta_{p}^{2}$ = 0.03, meaning that differences in sequence learning between age groups were not diverging between tasks, see **Table 1**.

**Table 1:** Sequence learning effects (random Block 11 subtracted from the mean of sequenced Blocks 10 and 12) in single, dual tone, and dual shape conditions for young and aged adults, derived from z-score transformed and untransformed RTs.

	Untransformed		Transformed	
	Young	Aged	Young	Aged
	Mean (SD)	Mean (SD)	Mean (SD)	Mean (SD)
Single	73 (58)	54 (35)	1,07 (0,85)	0,76 (0,50)
Dual tone	26 (32)	16 (38)	0,52 (0,64)	0,13 (0,32)
Dual shape	37 (40)	19 (29)	0,82 (0,96)	0,30 (0,46)

It is important to notice that when we carried out a repeated measures ANOVA to investigate sequence learning with Task Condition (single, dual tone, and dual shape) and Group (young and aged adults) as between-subjects factors, Sequence Learning (random Block 11 versus the mean of Blocks 10 and 12) as within-subjects factor, but without Explicit Awareness as a covariant, the Sequence Learning × Task Condition interaction effect became significant *F*(2,84) = 6.13, *p* < 0.01, $\eta_{p}^{2}$ = 0.13. This demonstrated a significant difference in sequence learning under single compared to dual tone conditions (*p* = 0.003), apparently modulated by explicit awareness. Other comparisons were not significant (*p* > 0.10).

To further investigate sequence learning, we performed the analysis separately for young and aged adults.

#### Young

A repeated measures ANCOVA on z-score transformed RTs was conducted with Task Condition (single, dual tone, and dual shape) as between-subjects factor, Sequence Learning (random Block 11 versus the mean of Blocks 10 and 12) as within-subjects factor, and Explicit Awareness as a covariate. There was no significant main effect of Task Condition, *F*(2,41) = 1.69, *p* = 0.20, $\eta_{p}^{2}$ = 0.08*.* The covariate, Explicit Awareness, tended to be related to Sequence Learning, *F*(1,41) = 3.88, *p* = 0.06, $\eta_{p}^{2}$ = 0.09. Sequence Learning was also significant, *F*(1,41) = 22.66, *p* < 0.001, $\eta_{p}^{2}$ = 0.36. More crucial is that the Sequence Learning × Task Condition interaction effect was not significant, demonstrating that after controlling for Explicit Awareness no differences between task conditions could be observed, *F*(2,41) = 0.44, *p* = 0.65, $\eta_{p}^{2}$ = 0.02.

#### Aged

A repeated measures ANCOVA on z-score transformed RTs was conducted with Task Condition (single, dual tone, and dual shape) as between-subjects factor, Sequence Learning (random Block 11 versus the mean of Blocks 10 and 12) as within-subjects factor, and Explicit Awareness as a covariate. There was no significant main effect of Task Condition, *F*(2,30) = 0.28, *p* = 0.76, $\eta_{p}^{2}$ = 0.02*.* The covariate, Explicit Awareness, was not significantly related to Sequence Learning, *F*(1,30) = 2.60, *p* = 0.12, $\eta_{p}^{2}$ = 0.08. Sequence Learning was significant, *F*(1,30) = 14.85, *p* = 0.001, $\eta_{p}^{2}$ = 0.33. Crucially, the Sequence Learning × Task Condition interaction effect remained significant after controlling for Explicit Awareness, *F*(2,30) = 7.70, *p* < 0.01, $\eta_{p}^{2}$ = 0.34. A *post hoc* Bonferroni test showed that learning was higher under the single task condition (*M* = 0.76, SD = 0.50) compared to the dual tone condition (*M* = 0.13, SD = 0.32, *p* = 0.001), and the dual shape condition (*M* = 0.30, SD = 0.46, *p* < 0.05). In addition, sequence learning was only significant in the single condition [*F*(1,8) = 15.93, *p* < 0.01, $\eta_{p}^{2}$ = 0.67], and showed a tendency toward significance in the dual shape condition, *F*(1,13) = 3.14, *p* = 0.10, $\eta_{p}^{2}$ = 0.20. However, it was no longer significant in the dual tone condition, *F*(1,7) = 0.59, *p* = 0.47, $\eta_{p}^{2}$ = 0.08.

#### Sequence learning: introduction versus recovery from interference

We disentangled the sequence learning effect by calculating sequence learning as measured by introducing random block interference (SL_Introduction_ = Block 10 vs. random Block 11) or by recovery from random block interference (SL_Recovery_: Block 12 vs. random Block 11). This could give us more insight in the underlying mechanism of impaired sequence learning and potential age differences. So, we carried out a repeated measures ANCOVA on z-transformed RTs with Task Condition (single, dual tone, and dual shape) and Group (young and aged adults) as between-subjects factors, Introduction/Recovery (SL_Introduction_ vs. SL_Recovery_) as within-subjects factor, and Explicit Awareness as a covariate. Results indicated that participants showed a tendency to acquire more sequence-specific knowledge in SL_Introduction_ as compared to SL_Recovery_ (main effect Introduction/Recovery, *F*(1,72) = 3.34, *p* = 0.07, $\eta_{p}^{2}$ = 0.04), which additionally demonstrated to be different between Task Conditions (interaction effect Introduction/Recovery × Task condition, *F*(2,72) = 5.88, *p* < 0.01, $\eta_{p}^{2}$ = 0.14). Planned contrasts showed that, when compared to the single condition, the difference between SL_Introduction_ and SL_Recovery_ was larger for the dual tone (*p* = 0.03), but not for the dual shape (*p* = 0.35). Under single task conditions SL_Introduction_ (*M *= 1.02, SD**= 0.76) was significantly higher than SL_Recovery_ (*M *= 0.84, SD**= 0.81), while in the dual tone condition SL_Introduction_ (*M *= 0.14, SD**= 0.71) was significantly lower than SL_Recovery_ (*M *= 0.52, SD**= 0.55). Other interaction effects were not significant, *p* > 0.10.

### COUNTING PERFORMANCE

A univariate ANOVA with Task Condition (dual tone and dual shape) and Group (young and aged adults) as between-subjects factors and counting performance as dependent factor, revealed no significant main effect of Task Condition, *F*(1,56) = 11.74, *p* = 0.18, $\eta_{p}^{2}$ = 0.92, nor a main effect of Group, *F*(1,56) = 0.77, *p* = 0.54, $\eta_{p}^{2}$ = 0.44. The interaction Task Condition × Group was also not significant, *F*(1,56) = 0.75, *p* = 0.39, $\eta_{p}^{2}$ = 0.01. [young: dual shape (*M* = 1.73%, SE = 0.46), and dual tone (*M* = 2.88%, SE = 0.43); aged: dual shape (*M* = 1.67%, SE = 0.64), and dual tone (*M* = 3.77%, SE = 0.63)].

## GENERAL DISCUSSION

In the current study, we investigated (1) the impact of a secondary task on implicit learning in young and aged adults, and (2) whether secondary tasks with varying interference levels differentially affect learning of an implicit sequence. Implicit learning effects were smaller in aged compared to young adults, possibly indicating that cognitive aging has an impact on implicit learning. Learning differences between single and dual task conditions disappeared for young adults when we accounted for explicit awareness. This effect can be expected as explicit knowledge leads to exaggerated knowledge in the single task condition**(Cleeremans et al., 1998). However, explicit awareness cannot be held responsible for the declined sequence learning under both dual task conditions (dual shape and dual tone) for aged adults. Further analysis indicated that, although the difference between learning under dual tone and dual shape conditions was not significant, sequence learning still tended to significance in the high interference condition (dual shape), while learning in the low interference condition (dual tone) diminished completely in aged adults. After analyzing sequence learning derived from introducing a random block compared to recovery from a random block, we can conclude that the diminished learning effect in the dual tone condition is due to a smaller learning effect after recovering from interference. Possibly, subjects in the dual tone condition are more cautious after the introduction of a random block, resulting in less sequence learning.

Contrary to our expectations, we found that in aged adults implicit learning was completely diminished in the low interference condition (dual tone), while in the high interference condition (dual shape) reduced learning could still be observed. Following these results, we could question whether the shape counting task was indeed more interfering than the tone counting task. Our a priori assumptions regarding the level of interference were based on cross modality and serial processing, where cross modal presentation (Duncan et al., 1997) and serial processing would be considered as less interfering (here the dual tone condition). Analyzing general RTs also showed a steeper decline between dual task Block 9 and single task Block 10 for the dual shape condition, meaning that subjects significantly gained speed when the dual shape task was eliminated. This could indicate that the dual shape task was experienced as highly interfering, which is in agreement with our a priori assumptions. Of course, also other factors such as the regularity embedded in the secondary task (e.g., Hemond et al., 2010; Nemeth et al., 2011) as well as timing of the secondary task stimuli (Hsiao and Reber, 2001) could affect the performance in the primary task. For example, the dual tone condition could also cause severe interference as secondary stimuli were not primarily associated with the relevant task, thereby disrupting sequence learning (Jiménez and Vazquez, 2005). Since young adults do not show differential learning patterns under high and low interference, we hypothesize that aged adults are more susceptible to these temporal disorganization effects. It could also be that aged adults have more difficulties in processing cross modal information, putting more pressure on their implicit learning skills. Further research should attempt to disentangle temporal disorganization effects and cross modal interference in implicit dual task learning in aged adults. For instance, a third condition could be included presenting the secondary task in the same modality (for example shape counting), but by presenting stimuli in such a way that the counting task will disrupt the timing of sequenced events (e.g., during the RSI). Comparing these results to the cross modal condition could demonstrate the importance of both cross modal interference and temporal disorganization effects in implicit dual task learning in aged adults.

Although interference seemed to play an important role in implicit learning under single and dual task conditions, we cannot disregard its interaction with cognitive aging. This age-related decline is more prominent in memory and learning tasks, affecting processing speed and executive functioning (Craik and Byrd, 1982; Stoltzfus et al., 1996; Park and Schwarz, 2000). Dual task coordination depends on the availability of cognitive resources, and is therefore of particular interest in aging studies. For example, when we would only analyze the results of young adults, it should be fair to conclude that implicit sequence learning is rather independent of attentional demands because there were no observed differences in learning between single and dual task conditions, even after controlling for explicit knowledge. However, aged adults demonstrated an implicit learning effect in the single task condition, while learning under dual shape condition proved to be reduced and learning under dual tone conditions was completely diminished. In addition, sequence learning was smaller for aged compared to young adults in general, presumably as an effect of cognitive aging. Therefore, we should point out that attentional demands apparently do have an influence on implicit learning. Possibly, the secondary task depleted the pool of cognitive resources leaving young adults with still enough capacity to learn, while aged adults only have a limited pool of resources at their disposal due to cognitive aging. Unfortunately, we have not included neuropsychological screening to pinpoint specific deficits in cognition and investigate the availability of cognitive resources. This needs to be taken into consideration for future studies by disentangling key cognitive processes lying on the basis of dual task performance. For example, it might be the ability to adequately inhibit irrelevant information, the efficiency to switch tasks, or perhaps a combination of different aspects regarding mental flexibility that are important for implicit learning under dual task conditions. It is important to provide insight in dual task learning by examining these cognitive processes, as it could also be extended to clinical populations expressing difficulties in implicit learning under attentional demands (e.g., freezing of gait in Parkinson’s disease, Vandenbossche et al., 2013).

To conclude, young adults showed higher implicit learning effects in general, and in aged adults dual task learning was significantly lower compared to the single task, even after controlling for explicit knowledge. This is in agreement with the hypothesis that attentional demands hamper implicit learning skills. However, aged adults were still able to learn an implicit sequence under dual task conditions as long as the secondary task shared relevant features with the primary task and was presented in the same modality. The present study extended the conclusions postulated by Frensch et al. (1998) stating that implicit learning is modulated by attention-based conditions in aged adults.

## AUTHOR CONTRIBUTIONS

Jochen Vandenbossche: Conception, Design, Execution, Writing of the paper; Daphné Coomans: Design, Review, and Critique; Koen Homblé: Design, Review, and Critique; Natacha Deroost: Conception, Design, Review, and Critique.

## Conflict of Interest Statement

The authors declare that the research was conducted in the absence of any commercial or financial relationships that could be construed as a potential conflict of interest.

## Footnotes

1 We calculated a z-score transformation of each participant’s RT by taking the median from a given block for an individual, subtracting his/her overall mean of all blocks, and dividing by the overall standard deviation of those block medians. This process was repeated for every participant and for every condition.

## References

[B1] Cleeremans A. Destrebecqz A. Boyer M. (1998). Implicit learning: news from the front. *Trends Cogn. Sci.* 2 406–414 10.1016/S1364-6613(98)01232-7 21227256

[B2] Coomans D. Deroost N. Zeischka P. Soetens E. (2011). On the automaticity of pure perceptual sequence learning. *Conscious. Cogn.* 20 1460–1472 10.1016/j.concog.2011.06.009 21741273

[B3] Craik F. I. Byrd M. (1982). “Aging and cognitive deficits,” in *Aging and Cognitive Processes* (New York, NY: Plenum Press) 191–211 10.1007/978-1-4684-4178-9_11

[B4] Curran T. (1997). Effects of aging on implicit sequence learning: accounting for sequence structure and explicit knowledge. *Psychol. Res.* 60 24–41 10.1007/BF00419678 9225617

[B5] Deroost N. Vandenbossche J. Zeischka P. Coomans D. Soetens E. (2012). Cognitive control: a role for implicit learning? *J. Exp. Psychol. Learn. Mem. Cogn.* 38 1243–1258 10.1037/a0027633 22428719

[B6] Destrebecqz A. Cleeremans A. (2001). Can sequence learning be implicit? New evidence with the process dissociation procedure. *Psychon. Bull. Rev.* 8 343–350 10.3758/BF03196171 11495124

[B7] Duncan J. Martens S. Ward R. (1997). Restricted attentional capacity within but not between sensory modalities. *Nature* 387 808–810 10.1038/42947 9194561

[B8] Faust M. E. Balota D. A. Spieler D. H. Ferraro F. R. (1999). Individual differences in information processing rate and amount: implications for group differences in response latency. *Psychol. Bull.* 125 777–799 10.1037/0033-2909.125.6.777 10589302

[B9] Frensch P. A. Lin J. Buchner A. (1998). Learning versus behavioral expression of the learned: the effects of a secondary tone-counting task on implicit learning in the serial reaction task. *Psychol. Res.* 61 83–98 10.1007/s004260050015

[B10] Frensch P. A. Miner C. S. (1994). Effects of presentation rate and individual differences in short-term memory capacity on an indirect measure of serial learning. *Mem. Cognit.* 22 95–110 10.3758/BF03202765 8035689

[B11] Gaillard V. Arnaud D. Michiels S. Cleeremans A. (2009). Effects of age and practice in sequence learning: a graded account of ageing, learning, and control. *Eur. J. Cogn. Psychol.* 21 255–282 10.1080/09541440802257423

[B12] Gamble K. R. Howard J. H. Jr. Howard D. V. (2014). Does a simultaneous memory load affect older and younger adults’ implicit associative learning? *Aging Neuropsychol. Cogn.* 21 52–67 10.1080/13825585.2013.782998 PMC3720785 23581975

[B13] Hedden T. Gabrieli J. D. (2004). Insights into the ageing mind: a view from cognitive neuroscience. *Nat. Rev. Neurosci.* 5 87–96 10.1038/nrn1323 14735112

[B14] Hemond C. Brown R. M. Robertson E. M. (2010). A distraction can impair or enhance motor performance. *J. Neurosci.* 30 650–654 10.1523/JNEUROSCI.4592-09.2010 20071529 PMC2823087

[B15] Howard D. V. Howard J. H. Jr (1992). Adult age differences in the rate of learning serial patterns: evidence from direct and indirect tests. *Psychol. Aging* 7 232–241 10.1037/0882-7974.7.2.232 1610513

[B16] Howard J. H. Jr. Howard D. V. (1997). Age differences in implicit learning of higher order dependencies in serial patterns. *Psychol. Aging* 12 634–656 10.1037/0882-7974.12.4.634 9416632

[B17] Hsiao A. T. Reber A. S. (2001). The dual-task SRT procedure: fine-tuning the timing. *Psychon. Bull. Rev.* 8 336–342 10.3758/BF03196170 11495123

[B18] Jiménez L. Méndez C. (1999). Which attention is needed for implicit sequence learning? *J. Exp. Psychol. Learn. Mem. Cogn.* 25 236–259 10.1037/0278-7393.25.1.236

[B19] Jiménez L. Vazquez G. A. (2005). Sequence learning under dual-task conditions: alternatives to a resource-based account. *Psychol. Res.* 69 352–368 10.1007/s00426-004-0210-9 15856287

[B20] Kürten J. De Vries M. H. Kowal K. Zwitserlood P. Flöel A. (2012). Age affects chunk-based, but not rule-based learning in artificial grammar acquisition. *Neurobiol. Aging* 33 1311–1317 10.1016/j.neurobiolaging.2010.10.008 21093109

[B21] Midford R. Kirsner K. (2005). Implicit and explicit learning in aged and young adults. *Aging Neuropsychol. Cogn.* 12 359–387 10.1080/13825580500246894 28486833

[B22] Nejati V. Garusi Farshi M. T. Ashayeri H. Aghdasi M. T. (2008). Dual task interference in implicit sequence learning by young and old adults. *Int. J. Geriatr. Psychiatry* 23 801–804 10.1002/gps.1976 18213607

[B23] Nemeth D. Janacsec K. Csifcsac G. Szvoboda G. Howard, Jr. J. H., Howard D. V. (2011). Interference between sentence processing and probabilistic implicit sequence learning. *PLoS ONE* 6:e17577 10.1371/journal.pone.0017577 PMC3050904 21408117

[B24] Nissen M. J. Bullemer P. T. (1987). Attentional requirements for learning: evidence from performance measures. *Cogn. Psychol.* 19 1–32 10.1016/0010-0285(87)90002-8

[B25] Park D. C. Schwarz N. E. (2000). *Cognitive aging: A primer.* Philadelphia: Psychology Press

[B26] Schneider W. Eschman A. Zuccolotto A. (2002). *E-Prime Version 1.1*. Pittsburgh: Psychology Software Tools.

[B27] Stadler M. A. (1995). Role of attention in sequence learning. *J. Exp. Psychol. Learn. Mem. Cogn.* 21 674–685 10.1037/0278-7393.21.3.674

[B28] Stoltzfus E. R. Hasher L. Zacks R. T. (1996). Working memory and aging: current status of the inhibitory view. *Work. Mem. Hum. Cogn.* 66–88

[B29] Vandenbossche J. Deroost N. Soetens E. Coomans D. Spildooren J. Vercruysse S. (2013). Impaired implicit sequence learning in Parkinson’s disease patients with freezing of gait. *Neuropsychology* 27 28–36 10.1037/a0031278 23356594

